# Mapping of the Gynoecy in Bitter Gourd (*Momordica charantia*) Using RAD-Seq Analysis

**DOI:** 10.1371/journal.pone.0087138

**Published:** 2014-01-30

**Authors:** Hideo Matsumura, Norimichi Miyagi, Naoki Taniai, Mai Fukushima, Kazuhiko Tarora, Ayano Shudo, Naoya Urasaki

**Affiliations:** 1 Gene Research Center, Shinshu University, Ueda, Nagano, Japan; 2 Okinawa Prefectural Agriculture Research Center, Itoman, Okinawa, Japan; Universidad Miguel Hernández de Elche, Spain

## Abstract

*Momordica charantia* is a monoecious plant of the Cucurbitaceae family that has both male and female unisexual flowers. Its unique gynoecious line, OHB61-5, is essential as a maternal parent in the production of F_1_ cultivars. To identify the DNA markers for this gynoecy, a RAD-seq (restriction-associated DNA tag sequencing) analysis was employed to reveal genome-wide DNA polymorphisms and to genotype the F_2_ progeny from a cross between OHB61-5 and a monoecious line. Based on a RAD-seq analysis of F_2_ individuals, a linkage map was constructed using 552 co-dominant markers. In addition, after analyzing the pooled genomic DNA from monoecious or gynoecious F_2_ plants, several SNP loci that are genetically linked to gynoecy were identified. GTFL-1, the closest SNP locus to the putative gynoecious locus, was converted to a conventional DNA marker using invader assay technology, which is applicable to the marker-assisted selection of gynoecy in *M. charantia* breeding.

## Introduction

Sexual reproduction systems in higher plants are highly divergent and vary depending on the plant adaptation to various environments. A majority of higher plants are hermaphrodite (bi-sexual) species, and approximately 6% of flowering plants are dioecious species [1] having separate male and female individuals. In addition to these sexual systems, monoecy, in which a plant carries both unisexual flowers (male and female in a single plant), is frequently observed in Cucurbitaceae species. Many well-known vegetable crops belong to the Cucurbitaceae, such as melon, cucumber squash and zucchini. *Momordica charantia* (bitter gourd, bitter melon) is also a monoecious Cucurbitaceae plant and is mainly cultivated in tropical and subtropical Asia. Sex determination in Cucurbitaceae has been studied in two major *Cucumis* species, melon (*C. melo*) and cucumber (*C. sativus*), and is regulated by ethylene [2]. In melon, two loci (*g* and *a*) determine the sex type. Plants carrying the dominant allele at both loci (*A-G-*) produce monoecious plants, whereas those with a recessive homozygosity at either the *g* or *a* locus (*A-gg* or *aaG-*) display gynoecy (all of the flowers are female) or andromonoecy (consisting of bi-sexual and male flowers), respectively [3]. Recessive homozygosity at both loci (*aagg*) results in hermaphroditic flowers [3]. The *A* gene encodes a 1-aminocyclopropane-1-carboxylic acid (ACC) synthase (CmACS-7) [4], and the *G* gene product is a zinc-finger transcriptional factor (CmWIP1) [5]. CmWIP1 has been suggested to repress carpel development together with the expression of *CmACS-7*. Because *CmACS-7* expression and the resultant ethylene production suppress stamen development, *CmACS-7* repression by *CmWIP1* leads to male flower development. In contrast, the inhibition of *CmWIP1* derepresses carpel development and *CmACS-7* expression, resulting in stamen repression and female flower development. In cucumber, sex determination has been suggested to be controlled by three genes, *F*, *A* and *M* [6], [7], [8]. The *F* gene is assumed to promote a female phenotype, and the *M* gene is responsible for maintaining monoecy. Furthermore, the homozygous recessive alleles of the *A* and *F* genes (*aaff*) cause androecy, indicating that the *A* gene is responsible for maleness. Based on the molecular cloning of the *F* and *M* genes, both of these genes encode ACC synthases (*CsACS1G* and *CsACS2*, respectively) [9], [10]. Although it remains unclear how ethylene biosynthesis is mediated by each of the ACC synthase genes, it has been speculated that the spatiotemporal regulation of ACC synthase could be important. A recent study indicated that the positive feedback regulation of *CsACS2* expression by ethylene [11] may be responsible for switching between female and male flower development.

In *M. charantia*, a gynoecious line (OHB61-5) is used in commercial F_1_ breeding as the maternal parent. Among *Momordica*, dioecious species such as *M. dioica* or *M. cochinchinensis* also exist. According to a molecular evolution study of *Momordica* spp., the genus originated from dioecious species in Africa, and seven reversions from dioecy to monoecy occurred during its dispersal to Asia [12], resulting in the diversification of monoecious and dioecious species. In dioecious *M. dioica*, the sex type is determined by a single locus [13]. The female carries a homozygous recessive allele, and the male is a heterozygote, leading to equal segregation ratio of males and females. Silver nitrate treatment converts females to hermaphrodite, indicating that ethylene signaling is also involved in sex determination [13]. Although knowledge of the genetic and molecular bases of sex determination is insufficient in monoecious *M. charantia*, *Momordica* species can be used to study the evolution of dioecy and monoecy.

Previously, another gynoecious line of *M. charantia* was reported (Gy263B) and was revealed to be under the control of a single recessive gene [14]. However, the specific causal gene was not identified. The objective of this study, therefore, was to genetically map the locus for gynoecy in OHB61-5 and identify DNA markers that are applicable to the selection of gynoecy in *M. charantia* breeding. Draft genome sequences of *M. charantia* are still unavailable, and its applicable DNA markers are limited [15], [16]. Therefore, a sequencing-based genotyping method has been employed as a rapid and efficient genetic mapping tool in this “non-model” plant species [17]. In the traditional genetic mapping approach using DNA markers such as SSR or AFLP, the advanced screening of polymorphisms among the parental lines was necessary for the identification of individual marker loci. Reference genome sequences are extremely useful for designing DNA markers such as genome-wide SNPs. Recently, next-generation sequencing (NGS)-based genotyping methods, including RAD-seq (restriction-associated DNA tag sequencing) [18] and GBS (genotyping by sequencing) [19] have been introduced as genetic mapping tools. These methods are based on sequencing of short fragments from defined positions in the genome and counting their frequency. DNA polymorphisms among cultivars or segregating individuals are represented by the presence or absence of these short sequences (tags). In contrast to whole-genome sequencing, the sequences of these short tags correspond to only a small portion of the genome. Nonetheless, these sequencing-based genotyping tools allow for the simultaneous identification of thousands of genome-wide polymorphisms.

In the present study, a RAD-seq protocol was modified for the efficient and high-throughput analysis of multiple samples. The protocol allowed for the rapid mapping of the gynoecious locus in *M. charantia*, and the conversion of one SNP that was linked to gynoecy to a conventional DNA marker that is applicable for practical marker-assisted selection in *M. charantia* breeding programs.

## Materials and Methods

### Plant Materials

A gynoecious line (OHB61-5) and a monoecious line (OHB95-1A) of *M. charantia* were stocked and grown at the Okinawa Prefectural Agricultural Research Center. The F_1_ plants were generated by crossing these two lines, and the F_2_ seeds were obtained by self-fertilization (pollination of female flowers with pollen from the same plant). The sex (male or female) of 20 flowers in each F_1_ or F_2_ plant was investigated. Plants carrying only the female flowers were defined as gynoecious plants, while the other plants were classified as monoecious plants in this study. Genomic DNA was extracted from a leaf of each plant using the DNeasy Plant Mini kit (Qiagen).

Seeds from plants in this study are available upon request to Hideo Matsumura. For OHB61-5, the F_2_ seeds from OHB61-5x OHB95-1A can be provided, owing to difficulty of its self-fertilizing seed production.

### Adapters for RAD-seq

Adapter-1 for PacI-digested DNA was prepared by annealing the two synthesized oligonucleotides 5′-biotin- GTACAGGTTCAGAGTTCTACAGTCCGACGATCXXXXXXAT-3′ and 5′-XXXXXXGATCGTCGGACTGTAGAACTCTGAACCTGfT-3 (XXXXXX correspond to the variable index sequences, Table S1). For AseI-digested DNA, the two oligonucleotides 5′-biotin- GTACAGGTTCAGAGTTCTACAGTCCGACGATCYYYYYY-3′ and 5′-TAYYYYYYGATCGTCGGACTGTAGAACTCTGAACCTGT-3′ (YYYYYY corresponds to the variable index sequences, Table S1) were synthesized and annealed. Adapter-2 was prepared by the annealing of two complementary oligonucleotides (5′-amino-CAAGCAGAAGACGGCATACGACATG-3′ and 5′-TCGTATGCCGTCTTCTGCTTG-3′).

The procedure for preparing these adapters is described in Methods S1.

### RAD-seq Analysis

A detailed protocol for the RAD-seq analysis is described in Methods S1.

Briefly, genomic DNA (100–300 ng) from individual plants or bulk samples was digested with PacI or AseI, which recognize TTAATTAA or ATTAAT, respectively. Biotinylated adapter-1, which was compatible with the digested ends, was ligated to the digested DNA fragments. After the elimination of the unligated adapters or adapter dimers, the adapter-1-ligated genomic DNA fragments were digested with NlaIII, which recognizes CATG. The biotinylated fragments were then collected using streptavidin-coated magnetic beads (Dynabeads M270, Dynal). Adapter-2, which is compatible with the NlaIII-digested end, was ligated to the fragments on the beads. After removing unligated adapter-2 by repeated washing, the adapter-ligated DNA on the beads was amplified by PCR using Phusion High-Fidelity DNA polymerase (Thermo Fisher Scientific) and the adapter primers (5′-AATGATACGGCGACCACCGACAGGTTCAGAGTTCTACAGTCCGA and 5′-CAAGCAGAAGACGGCATACGA). The pooled and purified PCR products were sequenced using the Illumina Genome Analyzer IIx or HiSeq2000 system. The sequencing primer was 5′-CGACAGGTTCAGAGTTCTACAGTCCGACGATC.

### RAD-tag Extraction

The CLC Genomics Workbench software (CLC bio) was used for processing the sequence reads (extraction of RAD-tags) and downstream analyses (data comparison). The sequence reads were classified based on the six-base indices for each sample (Table S1) and the 70-bp (for RAD-seq using PacI) or 95-bp (using AseI) sequences immediately after the index sequence were extracted as the tag. A list of tag sequences and their count was constructed for each sample. The procedure for tag extraction from the raw sequence data is described in Methods S2.

The sequence data in the present RAD-seq analysis are available in the DDBJ Sequence Read Archive (http://trace.ddbj.nig.ac.jp/dra/index_e.html) at accessions DRA001175, DRA001176, DRA001177, DRA001184 and DRA001185.

### Identification of Bi-allelic Tags as Markers

For identifying SNP (single nucleotide polymorphism) or SND (single nucleotide deletion) loci from the RAD-seq data using PacI, unique tags in either parental line (OHB61-5 or OHB95-1A) were first selected. The threshold for the selection of these tags was a count of more than fifty (>50x coverage) in one parent and a count of zero in the other parent. Then, the sequences of these OHB61-5-specific tags and the OHB-95-1A-specific tags were compared using the BLAST program. A pair of tags showing a single nucleotide difference was identified as a putative allele (bi-allelic tags) at the same locus. When more than two tags with single nucleotide differences were found, those tags were eliminated. These presumably polymorphic loci were employed in further study.

For the RAD-seq data using AseI, all of the analyzed tags were compared between the gynoecious F_2_ bulk and monoecious F_2_ bulk samples. Then, exclusively present tags with more than ten counts in the monoecious bulk samples were identified as the candidates for gynoecy-linked markers. Their allelic tags were searched by BLAST program against the RAD-seq data from OHB61-5. The identified bi-allelic tags carrying SNPs were employed as gynoecy-linked markers.

### Linkage Map Construction

The genotypes of the bi-allelic marker tags were determined in 48 F_2_ individuals (24 plants showing either the gynoecious or the monoecious phenotype) from a cross between OHB61-5 and OHB95-1A. For each locus (bi-allelic tag), the genotype was determined by the presence or absence of each allelic tag. When the tag appeared only once in the sample, it was regarded as a product of PCR or sequencing error and was not scored as present. The presence of both allelic tags at each locus represented a heterozygote, and the presence of either allelic tag was defined as a homozygote of the maternal or paternal allele.

This analysis was used to examine the segregation of each marker in 48 F_2_ plants, and the segregation ratio was evaluated as to whether these plants fit into a 1∶2:1 (OHB61-5-type homozygote:heterozygote:OHB95-1A-type homozygote) ratio. The genotype data of these bi-allelic tags in 48 F_2_ plants were analyzed using the AntMap program [20] to construct their linkage groups and linkage map.

### Genotyping of Gynoecy-linked RAD-seq Markers

For five selected gynoecy-linked marker loci (GTFL-1, 2, 3, 11 and 13), their 95-bp tag fragments were PCR-amplified from gynoecious F_2_ plants using primers that were designed based on the end sequence of each tag. The genotype (SNP type) of each locus was determined by the direct sequencing of the amplified fragments. Among the genotyped gynoecious F_2_ individuals, the number of heterozygous or OHB95-1A-type homozygous plants in each marker was scored. These plants derived from recombinant gametes between the gynoecious gene and each marker. The genetic distance (cM) between each marker locus and each putative gynoecious gene was calculated based on the frequency of these recombinant gametes.

### Primer Extension Capture (PEC) Analysis

The primer extension capture procedure was based on the method of Briggs et al. [21]. To optimize the capture of sequences that were adjacent to the RAD-tag, the protocol was modified and is described in Methods S3.

The genomic DNA from OHB61-5 was sonicated to prepare the capturing template, resulting in 300–500-bp fragments. The ends of the sheared DNA were repaired using NEBNext End Repair Module (New England Biolabs). An adapter from a TruSeq DNA sample prep kit (Illumina) was ligated to both ends of the fragments and amplified using primers from the same kit. The purified PCR fragments were used as the primary template DNA for the capture experiment.

Biotinylated oligonucleotides that were complementary to the GT1998 and GTFL-1 tag sequences (5′-bio-ATTAAATATGTATCGATATAGATATTTATTATCATTCTTGAG or 5′-bio-ATTAATCAATATTTTGCTCTTTACCTTGAG, respectively) were synthesized, respectively. The primary template DNA and biotinylated oligonucleotides were annealed, and primer extension was conducted using Phusion Hot Start High-Fidelity DNA polymerase (Thermo Fisher Scientific) at 98°C for 1 min and 60°C for 2 min. The reaction was immediately stopped by adding PB buffer from a MinElute PCR purification kit (Qiagen) followed by column purification using the same kit. The eluted DNA was bound to streptavidin-coated magnetic beads (Dynabeads M270, Dynal) at room temperature for 20 min and incubated at 70°C for 10 min. The beads were washed three times with hot (70°C) washing buffer (10 mM Tris-HCl, 1 mM EDTA and 1 M NaCl, pH 8.0) and TE buffer (10 mM Tris-HCl and 1 mM EDTA, pH 8.0) on a magnetic stand. The collected DNA fragments on the beads were PCR-amplified using adapter primers. The purified PCR products were used as the template DNA of a second capture, and the same experimental procedure was repeated.

The PCR product of the second capture was cloned into a plasmid followed by transformation into *E. coli* and plating. The insert of each transformant was amplified, and the fragment carrying the given tag sequence was screened via sequencing analysis.

### Invader Assay

The invader assay for the detection of SNPs at GTFL-1 was performed according to the procedure provided by Hologic (www.invaderchemistry.com). Oligonucleotides complementary to the target sequence that carried a non-matching base (an SNP at their ends) were synthesized as the invader oligo. In addition, two allele-specific signal probes that were complementary to the SNP region with arm sequences specific to the FRET cassettes were designed as described by Olivier [22]. The probe mix solution contained 2.5 µM signal probe (5′-CGCGCCGAGGAGTTGAGACATATAAATGCTTTC-3′amino and 5′-ACGGACGCGGAGGGTTGAGACATATAAATGCTTT-3′amino) and 0.25 µM Invader oligo (5′-CCTTGAGCTATGAACCCCTCGT). The target region, which included GTFL-1, was amplified from each F_2_ plant using the specific primer set (5′-AATTGCCTATAAGAAACCCTGTC and 5′-ATGAGAGCATGGTCATCGCAAG), and the PCR product was diluted after denaturing by incubation at 99°C for 10 min.

The invader reaction was initiated after adding the probe mix solution, cleavase and a FRET mix containing a fluorophore (6-FAM or Redmond Red) at the 5′ end and an internal quencher molecule. After incubation at 63°C for 40 min, the fluorescence of each solution was measured using a fluorescence microplate reader (ARVO X2, PerkinElmer). To detect 6-FAM fluorescence, 485-nm and 535-nm filters were used for excitation and emission, respectively. Redmond Red was measured using a 544-nm wavelength for excitation and 616-nm wavelength for emission.

The protocol for these analyses is described in Methods S4.

## Results

### Inheritance of Gynoecy in OHB61-5

OHB61-5 is a unique gynoecious bitter gourd line (*Momordica charantia*) from Okinawa that blooms only female flowers. To confirm the inheritance of its gynoecy, the OHB61-5 line was crossed with a monoecious line (OHB95-1A) that displays an approximately 5% frequency of female flowers per plant. By counting the number of male or female flowers in each F_1_ plant, the frequency of their female flowers was determined to be approximately 30%, defining these plants as monoecious plants. The sex type of the flowers in the offspring (F_2_) was similarly investigated, and the phenotype of these plants was determined to be either monoecious or gynoecious. We defined a gynoecious plant as having all female flowers, while all other plants were classified as monoecious. In the analyzed F_2_ population, the monoecious and gynoecious individuals were segregated in a 3∶1 (monoecious:gynoecious) ratio at a significant level (P>0.05 in the chi-square test, Table 1). These results suggest that gynoecy in OHB61-5 is determined by a single recessive gene.

**Table 1:** Segregation of gynoecy and monoecy in F_2_ population from OHB61-5x OHB95-1A.

Total analyzed F_2_ plants	Monoecious plants	Gynoecious plants	P-value of the chi-square test (3∶1)
49	37	12	0.96

### RAD-seq Analysis of Parental Lines

For the genetic mapping studies in *M. charantia*, the available DNA markers were limited. We, therefore, employed a RAD-seq analysis as a high-throughput tool for DNA polymorphism detection and genotyping. To improve the analytical throughput of this method, we modified the experimental steps in the original RAD-seq protocol [18]. First, we attempted to select appropriate restriction enzymes for digesting genomic DNA in higher plants. The frequency of restriction sites in the genome is a critical factor in the RAD-seq analysis because this frequency directly represents the number of detectable polymorphisms. According to an *in silico* survey of several plant reference genome sequences, the PacI or AseI sites carrying AT-rich sequences are more frequently observed than those restriction sites with G/C sequences (Figure S1). Both PacI and AseI are methylation insensitive and their fidelity of site recognition was confirmed in preliminary experiments. Thus, reproducible genotype data could be obtained. Second, the physical DNA shearing procedure was replaced by NlaIII digestion, which could be used to adjust the fragment size of the adapter-ligated DNA to make it more suitable for Illumina sequencing. This improvement facilitated the completion of the procedures for sample preparation, i.e., from genomic DNA to sequencing-ready fragments, in a PCR plate format. To sequence fragments from multiple samples, indexed adapters were allocated to each sample as previously described [23] (Table S1). Consequently, the present RAD-seq protocol was optimized for a high-throughput genotyping analysis.

The genomic DNA polymorphisms between two parental lines of *M. charantia* (OHB61-5 and OHB95-1A) were surveyed using the modified RAD-seq protocol with PacI. The sequence reads (76 bp) that were obtained using an Illumina Genome Analyzer IIx were separated using the six-base index sequences at the end of the adapters (Table S1), and the remaining 70-bp sequences with a PacI-digested end were extracted as the tag. The tag counts were calculated for each sample (Table 2). In total, 7,176,628 and 7,185,316 tags were obtained from OHB61-5 (gynoecious) and OHB95-1A (monoecious) plants, respectively. By comparing the tag sequences and counts between the two lines, the tags that were unique to one line, corresponding to DNA polymorphisms, were defined as either OHB61-5- or OHB95-1A-specific tags. The threshold of these parent-specific tags was a count of fifty (>50× coverage) in one parent and a count of zero in the other parent. Consequently, 1,183 and 1,318 unique tags were identified as OHB61-5- and OHB95-1A-specific tags, respectively (Table 2). By comparing the sequences of these unique tags between the two lines, 584 pairs of tags (loci) displayed SNP (single nucleotide polymorphism) or SND (single nucleotide deletion) (Table S2). These pairs of tags presumably represented alleles from single loci because they derived from a maternal-specific tag and a paternal-specific tag. These tags were designated as bi-allelic tags and were used as co-dominant markers to genotype the F_2_ population for further mapping studies.

**Table 2:** Summary of RAD-seq analysis of parental lines and their F_2_ population using PacI.

	Total count of analyzed tags (unique tags)	Parental line-specific unique tags*
OHB61-5	7,176,628 (156,644)	1,183
OHB95-1A	7,185,316 (137,920)	1,318
F_2_ (48 individuals)	122,829,622	–

* Tags, showing more than 50 count in either parental line but not present another parent.

### RAD-seq Analysis of the F_2_ Population

**Figure 1:**
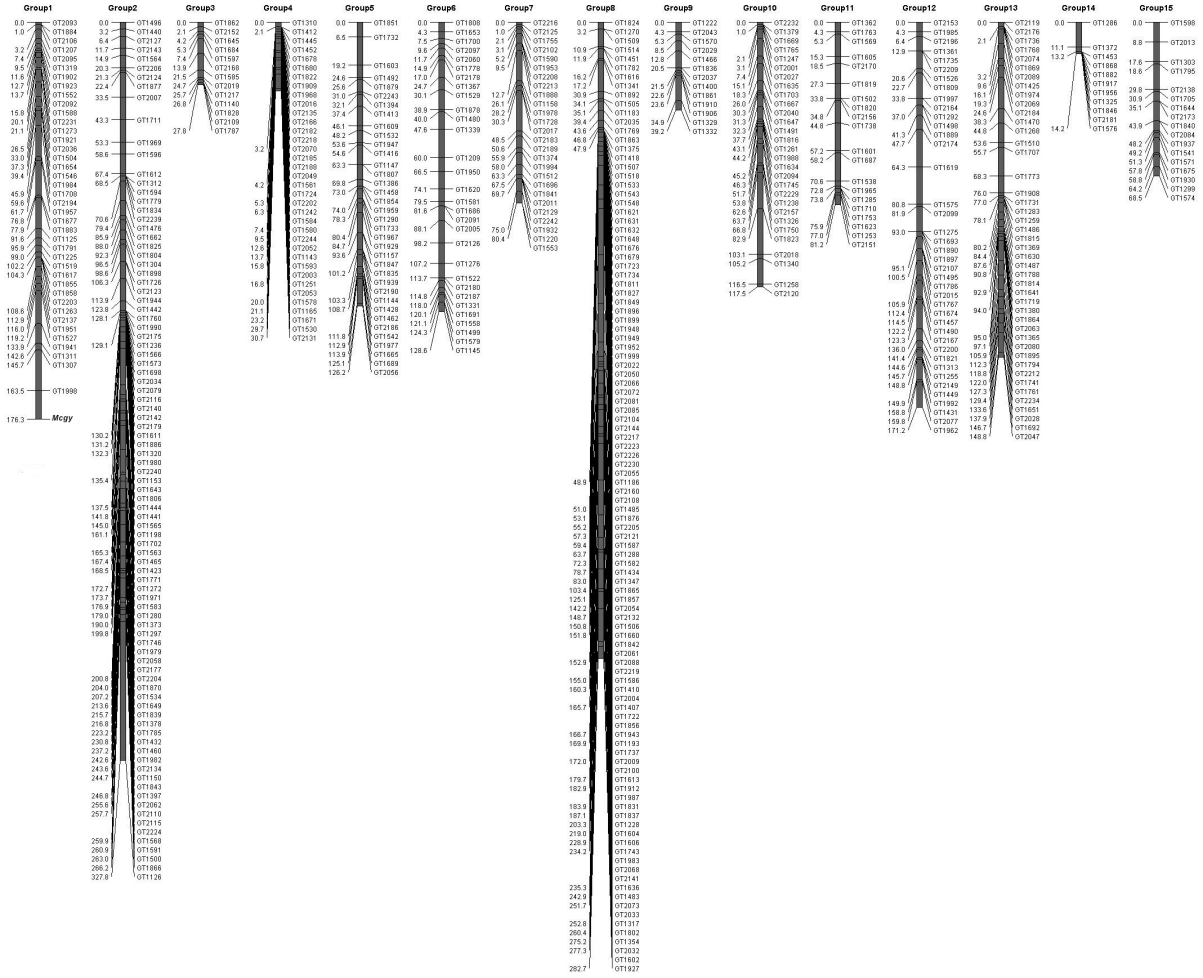
Linkage map in *M. charantia*. A linkage map was constructed using the AntMap program [20] by genotyping 552 co-dominant RAD-tag markers in 48 F_2_ individuals from the OHB61-5 and OHB95-1A lines. The sequences of each marker are shown in Table S2, and the genetic distances among the markers in each linkage group are listed in Table S4.

Of the F_2_ progeny that derived from the cross between OHB61-5 and OHB95-1A, 24 monoecious and 24 gynoecious plants were used for the RAD-seq analysis (Table S1). A total of 122,829,622 sequenced RAD-tags were obtained (Table 2), representing 2,558,950 tags per plant on average. The genotypes of the aforementioned 584 putative bi-allelic tags as markers were scored by the presence or absence of each tag in these F_2_ plants as described in the methods. Considering the error rate of PCR amplification and sequencing, it is possible that the tags that appeared only once in each F_2_ sample derived from an error of another allelic tag. Therefore, the tags that show more than two counts were defined as “present” for secure. In each bi-allelic tag (each locus), the presence of only maternal or paternal tags was used to define the homozygosity of either allele, and the appearance of both tags represented heterozygosity. In the entire analyzed F_2_ population, no plants lacked both allelic tags. According to the segregation in the analyzed bi-allelic tags in the F_2_ generation, most of these tags fit the expected segregation ratio (1∶2:1) except for 78 markers (Table S3). The genotype data of the analyzed tags in 48 F_2_ individuals were used in the genetic linkage analysis using the AntMap program [20]. Of the 584 markers, 552 markers were classified into 15 linkage groups by the grouping analysis under a LOD = 3.0 condition (Table S4), and the rests (32 markers) were ungrouped due to distinctive segregation. In addition, the marker loci in each linkage group were ordered based on the calculated genetic distances, and a genetic linkage map encompassing 1,821 cM was constructed (Figure 1).

On this linkage map, the putative gynoecious locus (*Mcgy*) was also mapped, according to phenotype of individual F_2_ plant (gynoecious or monoecious). This locus was located at the end of linkage group 1 (Figure 1), and the closest marker to *Mcgy* was GT1998, which comprised an allelic tag displaying an SNP between OHB61-5 (“C”-type) and OHB95-1A (“T”-type) (Table S2).

### Further Mapping Analysis of the Gynoecious Locus

**Figure 2:**
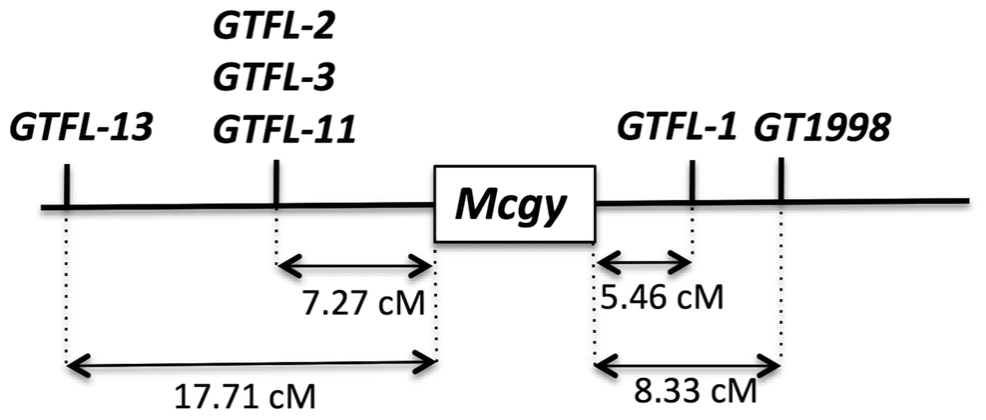
Genetic map of putative gynoecious locus (*Mcgy*). The genetic distances and location between each RAD-tag marker and the *Mcgy* locus were calculated from the genotypes of 55 gynoecious F_2_ plants. The sequences of alleles in these markers are shown in Table 4 (GTFL-1, GTFL-2, GTFL-3, GTFL-11, GTFL-13) and Table S2 (GT1998).

Because the number of linked markers and the genetic distance to the *Mcgy* locus in the above linkage map were still limited, additional closely linked DNA markers were investigated by the RAD-seq analysis using AseI, which has a six-base recognition site. In addition to the parental lines (OHB61-5 and OHB95-1A), the genomic DNA from gynoecious or monoecious F_2_ segregants was pooled (24 individuals in each pool) as gynoecious bulk or monoecious bulk samples and applied to the RAD-seq analysis. The prepared libraries were sequenced using an Illumina HiSeq2000 system, and a 95-bp sequence was extracted as a tag from each 101-base single-sequence read. In the parental lines, 11,240,886 and 14,792,025 tags were obtained from OHB61-5 and OHB95-1A, respectively, and the analyzed tags from the gynoecious and monoecious F_2_ bulk samples displayed 38,046,141 and 39,613,747 tags, respectively (Table 3). To identify the marker loci displaying linkage to gynoecy, we selected the tags that were exclusively present in the monoecious F_2_ bulk sample (>10 count) and the monoecious parent sample (OHB95-1A). Consequently, five unique tags were selected, and the predicted alleles of these five tags carrying SNPs were also found in OHB61-5. These five loci (bi-allelic tags) were designated as GTFL-1, GTFL-2, GTFL-3, GTFL-11 and GTFL13 (Table 4), and their genotypes (SNP-types) in the gynoecious F_2_ individuals were scored. The recombination rate between the *Mcgy* locus and each GTFL marker was calculated, and the genetic distance (cM) was estimated. In this analysis, we focused on the gynoecious F_2_ individuals as genotyping materials because monoecy was the dominant phenotype, and the genotypes of *Mcgy* locus (heterozygous or homozygous) were unknown in the monoecious F_2_ plants. Thus, the SNPs of these markers were scored in 55 gynoecious F_2_ individuals, comprising 24 plants that were used for the RAD-seq analysis and 31 additional gynoecious plants. The SNP type of each GTFL locus was determined by sequencing the 95-bp amplified product using a primer set that corresponding to the tag-end sequences (Table 4). From the genotype data of the GTFL markers, the genetic recombination rate between *Mcgy* and each GTFL marker was calculated. Consequently, a genetic map surrounding the *Mcgy* could be generated (Figure 2). The identified markers were distributed in the vicinity of the *Mcgy* locus, and the closest marker, GTFL-1, was located 5.46 cM from the *Mcgy*.

**Table 3:** Summary of RAD-seq analysis of parental lines and bulked F_2_ samples using AseI.

	OHB61-5	OHB95-1A	Gynoecious bulk	Monoecious bulk
Total count of analyzed tags	11,240,886	14,792,025	38,046,141	39,613,747

**Table 4:** RAD-tag markers using AseI, showing linkage to gynoecy in *M.charantia*.

			Tag count			
Marker ID	Tag sequence*	SNP**	Gynoecious F_2_	Monoecious F_2_	OHB61-5	OHB95-1A
GTFL-1	ATTAATCAATATTTTGCTCTTTACCTTGAGCTATGAACCCCTCGAGTTGAGACATATAAATGCTTTCTTCAAAAATTACCATTCAGAAAGGCAGT	“A”	**0**	**224**	**0**	**111**
	ATTAATCAATATTTTGCTCTTTACCTTGAGCTATGAACCCCTCGGGTTGAGACATATAAATGCTTTCTTCAAAAATTACCATTCAGAAAGGCAGT	“G”	**274**	**23**	**95**	**0**
GTFL-2	ATTAATGAGTCCGACTAAAATGGGTTTTTAAGTTAAGTAAATTAAAAATTTTGAAAGTTTTGTAAACCACTGAATTATTAGATTTCTAAATTCAA	“–”	**56**	**9**	**25**	**0**
	ATTAATGAGTCCGACTAAAATGGGTTTTTAAGTTAAGTAAATTAAAAATTTTGAAAGTTTTTGTAAACCACTGAATTATTAGATTTCTAAATTCA	“T”	**0**	**45**	**0**	**21**
GTFL-3	ATTAATGACTACCTAGGATTACACAGTTGAAAACACCCTCAATAAATCTACGAATATAGACTTTTTTCATACACTGCCCATAAACACTGAAGTCT	“A”	**0**	**38**	**0**	**13**
	ATTAATGACTACCTAGGATTACACAGTTGAAAACACCCTCAATAAATCTACGAATATGGACTTTTTTCATACACTGCCCATAAACACTGAAGTCT	“G”	**46**	**7**	**17**	**0**
GTFL-11	ATTAATAGATTGAAATTCTGTGTTCAGAGAAGCTGCACAAGAAACAAGTATCAGTATTCCTGAAGATTTTTATTGTTTTCTTGAACAAAAAAAGA	“A”	**0**	**19**	**0**	**10**
	ATTAATAGATTGAAATTCTGTGTTCAGAGAAGCTGCACAAGAAACGAGTATCAGTATTCCTGAAGATTTTTATTGTTTTCTTGAACAAAAAAAGA	“G”	**13**	**7**	**13**	**0**
GTFL-13	ATTAATTGCGTTGTCGAATTACATAGCTTACAGGCAAAATTGATCAATTTAGGAATTTCGGAGGAATAACGACGAGACGACAAAAGAAAACAGGG	“A”	**0**	**14**	**0**	**11**
	ATTAATTGCGTTGTCGAATTACATAGCTTACAGGCAAAATTGATCAATTTGGGAATTTCGGAGGAATAACGACGAGACGACAAAAGAAAACAGGG	“G”	**22**	**28**	**8**	**0**

* SNPs between parental lines in each marker were underlined in the tag sequences.

** SNP between OHB61-5 and OHB95-1A in each locus was indicated.

In addition, GT1998 derived from a previous RAD-seq analysis using PacI was allocated to the same genetic map surrounding the *Mcgy*. Because the tag size (70 bp) of GT1998 was not appropriate for PCR followed by direct sequencing, a longer genomic DNA fragment containing the tag was collected using a modified primer extension capture (PEC) method [21] (Methods S3). A 186-bp fragment containing GT1998 was obtained using extension capture (Figure S2A). By amplifying and sequencing this 186-bp fragment in 55 gynoecious F_2_ individuals, the SNP types at the GT1998 locus were determined. The genetic distance between the *Mcgy* and this position is indicated in the genetic map (Fig. 2). The GT1998 locus is located 8.33 cM from *Mcgy.*

### Invader Assay for the GTFL-1 Marker

**Figure 3:**
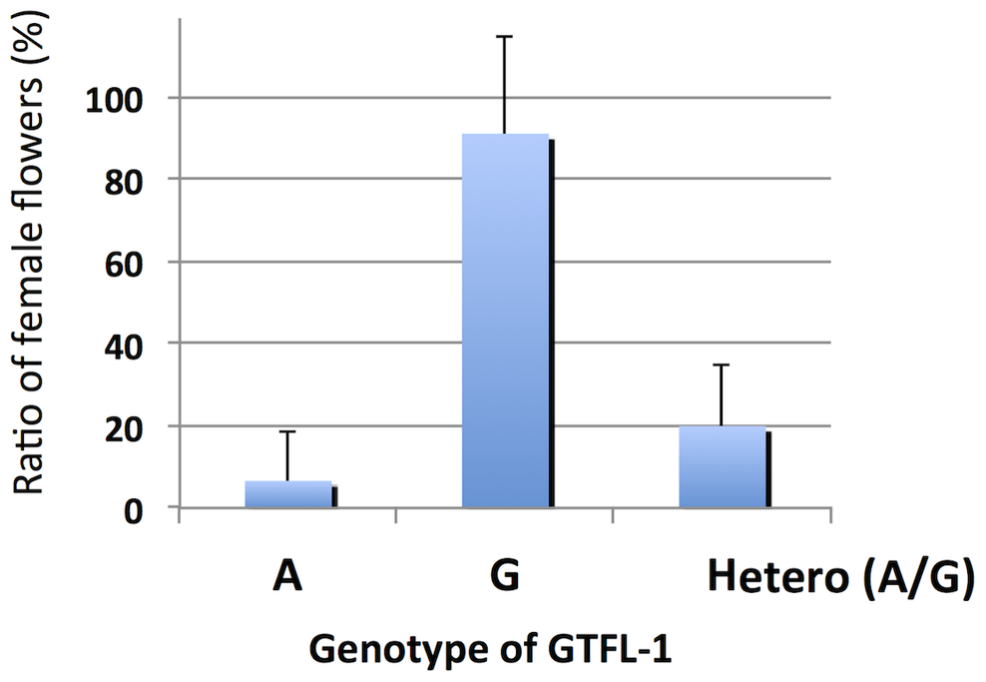
Female flower frequency and GTFL-1 genotype in F_2_ population. For 160 F_2_ plants from OHB61-5x OHB95-1A, the frequency of female flowers and GTFL-1 genotype in each plant was investigated, and the scoring results are shown in Table S5. The average frequency of female flowers (%) is indicated in the plants for each GTFL-1-genotype: “A” for the homozygotes of the OHB95-1A-type, “G” for the homozygotes of the OHB61-5-type and heterozygotes.

A conventional SNP-typing system was required for the high-throughput genotyping of numerous individuals when considering the application of the SNPs that were identified by RAD-seq to practical breeding programs for the development of new gynoecious lines in *M. charantia*. Therefore, we employed the invader assay system [22] as an SNP-typing tool for this purpose. GTFL-1, the closest SNP marker to *Mcgy*, was converted to an invader assay marker. First, the genomic fragment containing GTFL-1 was obtained by the PEC method (Methods S3), as the short 95-bp tag sequence was insufficient for designing probes for the assay. PCR primers, SNP (“G” or “A”) allele-specific signal probes and invader oligonucleotides were designed based on the sequence of the collected 502-bp fragment (Figure S2B). In the invader assay reaction, fluorescent signals are released by endonuclease cleavage (cleavase) of the allele-specific three-dimensional structures that are formed by annealing the target DNA, allele-specific signal probes and invader oligonucleotides. To detect each SNP allele, 6-FAM or Redmond Red was assigned to each signal probe. In the invader assay, the PCR-amplified target DNA was diluted once and incubated with the aforementioned probes, FRET mix and cleavase. Subsequently, the fluorescent signals of each sample were measured at the appropriate excitation and emission light for 6-FAM and Redmond Red. After the validation of the developed GTFL-1 invader marker using DNA from the parental lines (OHB61-5 and OHB95-1A), the marker was applied to assay the SNP type of 160 segregating F_2_ individuals (Table S5). Based on the signals of the two allocated fluorescent dyes, the “G” homozygotes (OHB61-5-type), “A” homozygotes (OHB95-1A-type) and heterozygotes (“G/A”) at GTFL-1 could be scored (Table S5). For the analyzed F_2_ plants, the genotype of the GTFL-1 locus segregated at 35∶83:42 (G/G:G/A:A/A). Simultaneously, the number of male and female flowers in each plant was also investigated in all of these 160 F_2_ plants (Figure S3, Table S5). The average frequency of female flowers per plant was 90.95% in the F_2_ plants carrying a homozygous “G” allele, including 29 gynoecious plants (Figure 3). In contrast, “A” homozygotes and heterozygotes at GTFL-1 displayed 6.6% and 19.92% female flower frequencies (Figure 3), respectively, and the differences were significant (P<0.05 in the t-test).

## Discussion

The RAD-seq analysis accelerated the detection of genome-wide polymorphisms in *M. charantia*, and genotyping in the F_2_ progeny was achieved using the same procedure in a high-throughput manner. Consequently, we were successful in the rapid genetic mapping of a gynoecious locus without any genomic resources such as a reference sequence, genetic map or known DNA markers. An identified SNP marker, GTFL-1, was linked to the gynoecious locus at a distance of 5.46 cM, indicating that GTFL-1 allows for the selection of gynoecious individuals among the segregating F_2_ progeny at >90% probability. In the present study, we succeeded in identifying several other gynoecy-linked markers. Therefore, applying these markers together with GTFL-1, marker-assisted selection will be more precise and efficient. Gynoecy is quite useful for producing F_1_ cultivars by avoiding self-pollination in the maternal parent. Gynoecy-linked markers, including GTFL-1, are useful for the rapid development of various gynoecious lines for disease resistance, fruit quality or yields.

Gynoecious plants are an effective material for elucidating sex determination in monoecious plants, as shown in *C. sativus* and *C. melo* [5], [9]. Gynoecy in *M. charantia*, as observed in OHB61-5, is determined by a single recessive gene, and hermaphrodite flowers are induced in this gynoecious line by silver nitrate treatment. These findings suggest that the sex determination of *M.charantia* is similar to that of *C. melo*, which is under the control of ethylene. Generally, ethylene is a phytohormone for senescence [24], fruit maturation [25] and cell expansion [26]. Therefore, it is unique that ethylene plays an important role in sex determination in Cucurbitaceae. However, it remains unclear how gaseous ethylene mediates the strict determination of floral organ development. Several approaches for identifying genes related to ethylene-mediated sex determination have been reported for melon and cucumber [27], [28], and other phytohormones, including auxin or gibberellin, independently affect sex in cucumber [29], [30]. However, the regulatory mechanisms that are involved are largely unknown. When comparing the identified genes for sex determination between melon and cucumber, the *A* gene in melon (*CmACS7*) and the *M* gene in cucumber (*CsACS2*) could be the counterparts for maintaining monoecious status. However, the genetic mechanism and identified genes (*CmWip1* and *CsACS1G*) for gynoecy are not always consistent between these two *Cucumis* species, implying a divergence of the sex determination pathway or responsible genes in Cucurbitaceae.

In addition to male or female differentiation in an individual flower, the ratio of male:female flowers in Cucurbitaceae crops is a crucial characteristic due to its influence on fruit yields. The phenotype of these plants is most likely genetically determined, but it is often affected by environmental conditions. The F_1_ cultivars of *M. charantia* typically display 50–60% female flowers per plant. In the present analysis, the frequency of female flowers was approximately 30% in the F_1_ plants of OHB61-5 and OHB95-1A, and their monoecious F_2_ progeny displayed distribution of female flower frequency ranging from 0% to 100% (Figure S3, Table S5). This result suggests that several loci participate in the determination of female flower frequency. Depending on the genotype at GTFL-1 in these monoecious F_2_ individuals, a slight but significantly higher female flower frequency was observed for heterozygotes (C/T) than for C-allele (monoecious parent type) homozygotes (Figure 3). This observation indicates that the gynoecious gene might have a semi-dominant effect on the sex ratio or that additional genes around the GTFL-1 locus could be responsible for the determination of the male-female ratio. As suggested in a previous study, the conversion between monoecy and dioecy occurs during the diversification of *Momordica* species [12]. In the analyzed F_2_ progeny, plants displaying no female flowers were observed and were considered to be displaying monoecy rather than androecy due to the limited number of scored flowers (20 flowers) per plant. However, these plants displaying an extremely biased sex ratio might convert from to dioecy from monoecy. The isolation of the gynoecious gene in OHB61-5 will contribute to understanding the regulation of sex ratio and its evolution in *Momordica* species.

RAD-seq and other sequencing-based genotyping methods have been applied to genetic mapping in several higher plant species [31], [32], [33], [34], [35]. Our protocol included two modified steps to increase the throughput. The first modification was the choice of an appropriate restriction enzyme for the plant genome analysis. In the original RAD-seq analysis, SbfI (CCTGCAGG) was used with stickleback or zebrafish [18], [36], because restriction sites are common in the genomes of these fish. However, SbfI would rarely cleave plant genomic DNA, as deduced from the reference genome sequences of several plant species (Figure S1). However, PacI and AseI sites are frequent in plant genomes. These enzymes are methylation insensitive, and thus, reproducible data were expected to be independent of differential methylation events among the samples. Second, the sharing of adapter-ligated genomic DNA fragments was substituted for trimming by NlaIII digestion. This change allowed for the reproducible and high-throughput preparation of RAD-tag fragments in a 96-well plate format. In addition, the present RAD-seq protocol facilitated the generation of sequencing-ready fragments from 48 F_2_ individuals with a reduced effort. Because the capability of current generation DNA sequencers is extremely high, an analysis of hundreds of samples can be accomplished within a few days.

In the RAD-seq data, DNA polymorphisms were represented by tags unique to either sample, including nucleotide substitutions or In/Dels. Unique tags that derived from substitutions or In/Dels of one to several nucleotides in the tag sequence were appropriate for genetic mapping as co-dominant markers as their allelic tags could be found. However, it was difficult to identify alleles without reference genome, when unique tags represent the In/Dels of whole tag sequences or polymorphisms at restriction sites in one parent. These representative tags are only applicable as dominant markers. Due to the absence of a reference genome sequence for *M. charantia*, sequence polymorphisms could not be identified by genome mapping of the RAD-tags. Instead, the sequences of unique tags in each parent (OHB61-5 or OHB95-1A) were compared to identify single nucleotide differences as bi-allelic tags. According to the segregation of these tags in the F_2_ population, more than 85% of the analyzed bi-allelic tags were confirmed to be segregated as expected (1∶2:1). The other tags might show a distorted segregation, as an equal number of recessive gynoecious plants and dominant monoecious plants in the F_2_ population were analyzed for genotyping. When two tags were derived from genetically independent loci, a few F_2_ plants (1/16 of 48 plants) lacking both tag sequences would be segregated. Those plants with missing tags were not observed in any markers. Therefore, the selected pairs of tags in the study could be allelic or genetically linked at least.

In our study, the number of linkage groups (15 groups) did not converge on a known chromosome number (11 chromosomes) in *M.charantia* [37], and some linkage groups carried only a limited number of markers. These results are most likely due to a bias of marker location in the analyzed population or the separation of some linkage groups on the same chromosome in the present linkage analysis. To address these problems, it will be necessary to increase the marker density or replace the mapping population. A RAD-seq analysis using additional different restriction enzymes will increase the probability of finding markers. A greater number of gynoecy-linked markers can be identified by RAD-seq analysis with AseI than with PacI. For the fine-scale mapping of a gynoecious locus, an analysis using four or five base recognition enzymes could be helpful as the GBS method [19]. With respect to the mapping population, an increase in the number of F_2_ individuals will contribute to the development of a precise linkage map. However, when the sequence divergence between the parents of a mapping population is less, the fine-scale mapping of the gynoecious gene by replacing the restriction enzymes or increasing the population size can be difficult. Therefore, an additional F_2_ population can be effective when developed via another monoecious parent that is genetically distant from the gynoecious OHB61-5.

As demonstrated in previous publications [38], RAD-seq analysis is an effective tool for genetic mapping, particularly in organisms lacking a reference genome. After narrowing down the target locus by mapping with the RAD-seq, the genotyping of selected individual polymorphisms is required for the fine-scale mapping of the target locus or marker-assisted breeding programs. Due to the improvement in the reliable sequence read length in Illumina sequencing, a sufficient size (95 bp) of RAD-tags for PCR amplification could be obtained, as demonstrated in this study, allowing for the genotyping of tags in F_2_ individuals by simple PCR and sequencing. However, this approach was not applicable to polymorphisms that were located at the end of the tag sequence. Primer extension capture [21] is helpful for designing PCR primers for amplifying and genotyping polymorphisms in any position in the RAD-tag. A combination of RAD-seq and PEC strongly supports the development of conventional DNA markers in any organisms without the requirement for known genome sequence data. A large-scale DNA fragment collection of specific genomic regions was previously established based on the sequence capture method [39], which employs the hybridization of oligonucleotides that are complementary to known genomic DNA sequences containing the target DNA fragments. Commercially available sequence capture tools allow for the collection of large genomic regions or exon regions of multiple genes in a high-throughput manner [40], and a reference genome sequence is required for this method. Briggs et al. [21] employed PEC to recover and reconstruct the mitochondrial genome from ancient DNA. Other than its current applications, the PEC protocol might be useful for gap-filling among *de novo* assembled scaffolds.

Because SNPs are the most abundant type of DNA polymorphism, it was inevitable that they would be considered for use as a practical DNA marker. Recent progress in sequencing technologies has allowed for the identification of a large number of SNPs. SNP-typing tools such as SNP chips [41] and bead arrays [42] are currently commercially available, although most of these tools are intended to simultaneously score thousands of SNP loci. These technologies are not applicable to genotyping a limited number of SNP loci. The invader assay is a well-known genotyping method for individual SNP markers [22] and is employed in clinical applications [43]. Our results demonstrated that this assay is applicable to higher plants. Technically, the invader assay has no critical caveats with respect to the rapid and accurate genotyping of SNP in hundreds of individuals. However, the high analytical cost per a sample might not be acceptable for the routine genotyping of marker-assisted selection used in crop breeding. In addition to the invader assay, TILLING (targeting induced local lesions in genomes) [44] and allele-specific PCR [45] are alternative methods for conventional SNP typing. However, the cost, throughput and accuracy of genotyping should be considered when selecting an appropriate SNP-typing method.

## Supporting Information

Figure S1 **Number of restriction sites in plant genome.**
(PPTX)

Figure S2 **Sequences of genomic DNA fragment comprising GT1998 tag (A) and GTFL-1 tag (B), obtained by primer extension capture.**
(DOCX)

Figure S3 **Distribution of F_2_ plants showing different female flower frequencies.**
(PPTX)

Table S1 **List of analyzed samples and allocated index sequences in the RAD-seq analysis using PacI.**
(XLSX)

Table S2 **List of putative bi-allelic tags between OHB61-5 and OHB95-1A.**
(XLSX)

Table S3 **Segregation of biallelic-tag markers in 48 F_2_ plants.**
(XLS)

Table S4 **Orders and Genetic distances among the co-dominant markers in each linkage group.**
(XLSX)

Table S5 **Female flower frequency and GTFL-1 genotype in 160 F_2_ plants from OHB61-5 x OHB95-1A.**
(XLSX)

Methods S1 **RAD-seq analysis experimental protocol.**
(DOCX)

Methods S2 **RAD-seq analysis data analysis protocol.**
(DOCX)

Methods S3 **Primer extension capture protocol.**
(DOCX)

Methods S4 **Invader assay protocol.**
(DOCX)
