# Supplementary material for: Mapping of the Gynoecy in Bitter Gourd (Momordica charantia) Using RAD-Seq Analysis
Source: PLoS One. 2014 Jan 30;9(1):e87138. doi: 10.1371/journal.pone.0087138 (PMC3907450; doi:10.1371/journal.pone.0087138)
Supplement: Figure S2 — Sequences of genomic DNA fragment comprising GT1998 tag (A) and GTFL-1 tag (B), obtained by primer extension capture. (DOCX) [file pone.0087138.s002.docx]

(A)

TATACAGTTTGAAATTGAAGATAGTATTTTAATCAATTTAATTAAATATGTATCGATATAGATATTTATTATCATTCTTGAGTTAAATAGATGATGACCAATTTTCTTTTTTTTTTTTAAACAACCGACAATAAATTTATACAAGGGTTGAATTCTGTTGATGTTAAAAATCTGCCTTTCAGAAAA

(B)

GCGTACTCTTTTATGTTTTTTTAAATATGTTTAAAATGAAATAATAAGAGACTAAATAAAAAAATATAATTGCCTATAAGAAACCCTGTCTAAGCAAGGTTCTGTCTTTGTTGGGGTACTTAAGTTGATCGTAAGAGAACACCTTTTCCTAAAAACCGATATGGGGGTTGAATTAATCAATATTTTGCTCTTTACCTTGAGCTATGAACCCCTCGGGTTGAGACATATAAATGCTTTCTTCAAAAATTACCATTCAGAAAGGCAGTCTTGACGTCCATTTGCCAATTCAACCCCGGGTTGAGCATGTTCTTGCATTGATGTTCCCTCTTTCATGCGGGAATTGTAAATGAACCGTAGGGCATTGTGTCGGGCTTGAATGGACGGCTGTCCAAACATGTCCTGCAACGAGTCCATGATCTTGCTTGCGATGACCATGCTCTCATGCTTCTTGGCCAGCATATTAGATATGCTTGCCAAGCTGTAAACCTTGGCCTTGTCAATG

Figure S2 Sequences of genomic DNA fragment comprising GT1998 tag (A) and GTFL-1 tag (B), obtained by primer extension capture.

RAD-tag sequence for GT1998 or GTFL-1 was underlined. Nucleotide of SNP position was surrounded by a square in each sequence. Both sequences represented genomic DNA from OHB61-5.
