# Supplementary material for: Mapping of the Gynoecy in Bitter Gourd (Momordica charantia) Using RAD-Seq Analysis
Source: PLoS One. 2014 Jan 30;9(1):e87138. doi: 10.1371/journal.pone.0087138 (PMC3907450; doi:10.1371/journal.pone.0087138)
Supplement: Methods S1 — RAD-seq analysis experimental protocol. (DOCX) [file pone.0087138.s009.docx]

**Methods S1**

RAD-seq analysis experimental protocol

1. Genomic DNA digestion with restriction enzymes

For the digestion, 100 ng genomic DNA was prepared and to it was added 10 µl 10x NEBuffer 1 (for *Pac*I) or 10x NEBuffer 3 (for AseI), 1 µl BSA (100 µg/ml), 2 µl PacI (10 u/µl) or *Ase*I (10 u/µl) and dH20 to a total volume of 100 µl.

The mixed solution was incubated at 37˚C for 2 hr.

1. Purification of digested DNA

For purifying the digested genomic DNA, 180 µl AMPure XP beads suspension was added to 100 µl digested DNA solution and mixed well by pipetting. The tube was allowed to stand for 5 min at room temperature before it was moved to a magnetic tube stand and allowed to stand for 5 min.

After the supernatant was clear, the clear solution was removed and discarded. Then, 200 µl 70% EtOH was added to the beads in the tube. After 30 sec, the supernatant was removed. This 70% EtOH washing step was then repeated. After removing the 70% EtOH, the beads were dried for approximately 2 min.

After the tube was removed from the magnetic stand, 23 µl TE buffer (10 mM Tris-HCl, 1 mM EDTA, pH 7.5) was added and mixed well by pipetting. The tube was placed on a magnetic stand and allowed to stand for 1 min. The clear supernatant was transferred to another tube as with the purified digested DNA solution.

1. Preparation of adapters

The oligonucleotide sequences for adapters are described in the method section. These oligonucleotides should be purified with HPLC or OPC (Oligonucleotide Purification Cartridge).

For annealing the oligonucleotides, 10 µl each sense and anti-sense oligonucleotides (each 100 pmol/µl) were mixed and combined with 3 µl 10× polynucleotide kinase buffer and 7 µL TE buffer. A total of 30 µl of this mixture was denatured at 95°C for 2 min and cooled down to 20°C for annealing the complementary oligonucleotides. The annealed double-stranded DNA fragments were designated adapter-1 and adapter-2, respectively..

1. Adapter-1 ligation

Before adapter-1 ligation, the allocation of indexed adapter-1 sequences to the samples was determined.

To purified digested genomic DNA (20 µl), 3 µl 10x T4 DNA ligase buffer, 0.5 µl adapter-1 solution, 2.5 µl dH2O and 4 µl T4 DNA ligase (5 u/µl) were added and mixed. The mixed solution was incubated at 16˚C for 2 hr.

1. Purification of adapter-1-ligated DNA

After the ligation reaction, 54 µl of the AMPure XP bead suspension was added to the reaction solution and mixed well. The procedure that is described in the above step 2 was followed.

1. NlaIII digestion of adapter-1-ligated DNA

For the *Nla*III digestion, 5 µl 10x NEBuffer 4, 0.5 µl BSA (100 µg/ml), 22.5 µl dH2O and 2 µl *Nla*III (10 u/µl) were added to 20 µl purified adapter-1-ligated DNA solution.

The mixed solution was incubated at 37˚C for 2 hr.

1. Binding to streptavidin-coated magnetic beads

A 50-µl Dynabeads C1 streptavidin bead suspension was prepared in a siliconized tube. The tube was placed on a magnetic stand for 1 min, and the clear supernatant was removed. The tube was then placed on a tube stand, and 100 µl 1x B&W (binding and washing buffer; 5 mM Tris-HCl, pH 7.5, 0.5 mM EDTA, 1 M NaCl) was added to the beads for washing. The beads were resuspended and placed on a magnetic stand. After 1 min, the clear supernatant was removed.

To the washed beads, 50 µl *Nla*III digestion solution and 50 µl 2x B&W (10 mM Tris-HCl, pH 7.5, 1 mM EDTA, 2 M NaCl) were added. The beads were suspended and incubated at room temperature for 20 min. The tube was then placed on a magnetic stand, and the supernatant was removed after 1 min.

The beads were washed with 1x B&W solution twice as described above and once washed with TE buffer. Finally, the beads were resuspended in 20 µl TE buffer.

1. Adapter-2 ligation

To the suspended beads solution, 3 µl 10x T4 DNA ligase buffer, 0.5 µl adapter-2 solution, 2.5 µl dH2O and 4 µl T4 DNA ligase (5 u/µl) were added and mixed. The mixed solution was incubated at 16˚C for 2 hr.

After the ligation reaction, the tube was placed on a magnetic stand for 1 min, and the supernatant was removed. The magnetic beads were washed with 1x B&W solution three times and with TE buffer three times as described in step 7. The washed beads were resuspended in 25 µl TE buffer.

1. PCR amplification of the adapter-ligated fragments

The adapter-ligated DNA fragments on the magnetic beads were amplified as follows. A PCR reaction mixture was prepared in a tube by adding 4 µl 5x Phusion HF buffer, 0.4 µl 50 mM MgCl2, 0.8 µl 2 mM dNTP, 0.2 µl each adapter primer (50 pmol/µl; their oligonucleotide sequences are described in the methods section), 12.5 µl dH2O, 2 µl magnetic bead suspension with immobilized adapter-ligated DNA, and 0.25 µl Phusion High Hot start DNA polymerase (2 u/µl).

　The PCR conditions were as follows:

98°C for 2 min

15-18 cycles of

98°C for 10 sec

55°C for 20 sec

72°C for 30 sec

subsequently

72°C for 5 minutes

Hold at 4°C.

The product from four-to-eight tubes of the same PCR reaction was collected and purified using AMPure XP as described above. Finally, 27 µl TE buffer was added to the beads, and 25 µl supernatant was transferred as purified DNA for sequencing.
