# Supplementary material for: Mapping of the Gynoecy in Bitter Gourd (Momordica charantia) Using RAD-Seq Analysis
Source: PLoS One. 2014 Jan 30;9(1):e87138. doi: 10.1371/journal.pone.0087138 (PMC3907450; doi:10.1371/journal.pone.0087138)
Supplement: Methods S2 — RAD-seq analysis data analysis protocol. (DOCX) [file pone.0087138.s010.docx]

**Methods S2**

RAD-seq analysis data analysis protocol

The separation by index sequences, tag extraction from raw sequence data and tag counting were carried out using the CLC Genomics Workbench software.

1. Separation by index and tag extraction

The raw sequence data (fastq) file was imported to the CLC Genomics Workbench.

The following sequence of selections was performed: Toolbox 🡪 High-throughput Sequencing 🡪Expression Profiling by Tags 🡪 Extract and Count Tags.

The imported fastq files were selected for tag extraction.

In next dialog, the employed index sequences (6-bases) were input as Sample keys, and the tag sequence length (70 or 90 bases in the present study) to be extracted was entered as the Sequence.

Subsequently, the raw counts as tabulated by the tags were selected.

For the output options, create expression samples with tag counts was selected.

The output file contained a list of the extracted tags and their counts in each indexed sample.

1. Comparison of tags among samples

The following sequence of selections was performed: Toolbox 🡪 Expression Analysis 🡪 Set Up Experiment.

The created tag and tag count file to be compared were selected.

The number of groups to be compared was defined. Usually the Unpaired comparison was selected.

The sample names were input and assigned to tag data files.

The created file including compared tags and their counts among samples was opened.

By filtering, the tags without appropriate digested end sequences were eliminated.

Additionally, any unique tags in a particular sample were extracted by filtering the tag count.
