# Supplementary material for: Mapping of the Gynoecy in Bitter Gourd (Momordica charantia) Using RAD-Seq Analysis
Source: PLoS One. 2014 Jan 30;9(1):e87138. doi: 10.1371/journal.pone.0087138 (PMC3907450; doi:10.1371/journal.pone.0087138)
Supplement: Methods S3 — Primer extension capture protocol. (DOCX) [file pone.0087138.s011.docx]

**Methods S3**

Primer extension capture protocol

1. Preparation of capturing template

The procedure for capturing the template DNA (adapter-ligated genomic DNA fragments) followed the protocol of a TruSeq DNA sample prep kit (Illumina), and solutions in the kit were used for the reaction.

Genomic DNA (1 µg) was fragmented by sonication to approximately 300-500 bp.

The ends of the fragmented DNA in 60 µl TE buffer were end-repaired by adding 40 µl End Repair Mix and incubating at 30 ˚C for 30 min.

To the 100 µl end-repaired DNA solution, 160 µl AMPure XP bead suspension was added. After incubation for 15 min, the solution was placed on a magnetic stand, and the supernatant was removed. Then, 200 µl 80% EtOH was added, and the supernatant was removed after 30 sec.

The 80% EtOH washing step was repeated twice.

The bead pellet was resuspended with 17.5 µl Resuspension buffer and placed on the magnetic stand. A 15-µl aliquot of the supernatant was transferred to a new tube.

To the purified DNA fragments 2.5 µl Resuspension buffer and 2.5 µl A-tailing Mix were added. The mixture was incubated at 37 ˚C for 30 min.

To the A-tailing reaction solution 2.5 µl Resuspension buffer, 2.5 µl DNA Ligase Mix and 2.5 µl DNA Adapter Index (one of the Index) were added. The mixture was incubated at 30 ˚C for 10 min. After adding 5 µl Stop Ligase Mix, 42.5 µl AMPure XP bead suspension was added to the solution to purify the adapter-ligated DNA.

The beads were washed following the procedure described above. The washed beads were resuspended in 22.5 µl Resuspension buffer, and 20 µl supernatant was removed as purified DNA after placing the tube on a magnetic stand.

For the amplification of the prepared DNA fragments, 5 µl PCR Primer Cocktail and 25 µl PCR Master Mix were added to 20 µl purified adapter-ligated fragment solution. The PCR conditions were as follows:

98°C for 30 seconds

10 cycles of

98°C for 10 seconds

60°C for 30 seconds

72°C for 30 seconds

subsequently

72°C for 5 minutes

Hold at 4°C.

For purifying the PCR amplified product, 50 µl AMPure XP bead suspension was added to the PCR solution. The beads were washed following the procedure described above. The washed beads were resuspended in 22.5 µl TE buffer, and 20 µl supernatant was taken as purified DNA after placing the tube on a magnetic stand.

This purified DNA was used as the capturing template.

1. Capturing reaction

The mixture for primer extension was prepared and contained the following:

5x Phusion HF buffer: 4 µl

50 mM MgCl2: 0.4 µl

2 mM dNTP: 0.8 µl

biotinylated oligonucleotide (100 pmol/µl): 0.2 µl

dH2O: 12.7 µl

capturing template: 2 µl

Phusion High Hot Start DNA polymerase (2 u/µl): 0.25 µl.

The mixture was incubated at 98 ˚C for 1 min and 60 ˚C for 2 min.

After finishing the above incubation, 100 µl PB buffer from the MinElute PCR purification kit (Qiagen) was immediately added to the PCR reaction solution.

The mixture was transferred to a MinElute column (Qiagen) and centrifuged at 13,000 rpm for 1 min. From the same kit, 750 µl PE buffer was added, and the column was centrifuged again. The solution was discarded and the column was centrifuged without adding a buffer for drying. The column was transferred to a new tube to which 15 µl TE buffer was added. The tube was centrifuged to collect the purified DNA after incubation for 1 min.

To the collected purified DNA solution, 15 µl 2x B&W buffer and 30 µl streptavidin-coated magnetic bead (Dynabeads M270) suspension were added.

The tube was incubated at room temperature for 20 min and at 70 ˚C for 10 min.

The tube was placed on the magnetic stand, and the supernatant was removed. Immediately, the beads were resuspended in 1x B&W buffer at 70 ˚C, and the supernatant was removed after placing the tube on a magnetic stand. The washing step was repeated twice. Then, the washing buffer was replaced with TE buffer at 70 ˚C, and the beads were washed twice.

To amplify the captured DNA on the beads, a PCR mixture was prepared as follows:

5x Phusion HF buffer: 4 µl

50 mM MgCl2: 0.4 µl

2 mM dNTP: 0.8 µl

PCR Primer Cocktail (TruSeq DNA sample prep kit (Illumina)): 2.5 µl

dH2O: 10.65 µl

bead suspension with captured DNA: 5 µl

Phusion High Hot start DNA polymerase (2 u/µl): 0.25 µl

Then, the DNA was amplified as follows:

98°C for 30 seconds

15 cycles of

98°C for 10 seconds

60°C for 30 seconds

72°C for 30 seconds

subsequently

72°C for 5 minutes

Hold at 4°C.

The PCR product was purified using AMPure XP beads as described above (in Preparation of capturing template). Finally, the product was dissolved in 20 µl TE buffer.

Using the purified PCR product from the captured DNA as the capturing template, the primer extension, streptavidin beads-capture, washing and PCR amplification procedure were repeated.

The collected purified DNA fragments (PCR product) from the second capturing reaction were cloned using Zero Blunt TOPO PCR cloning kit.
