# Supplementary material for: Mapping of the Gynoecy in Bitter Gourd (Momordica charantia) Using RAD-Seq Analysis
Source: PLoS One. 2014 Jan 30;9(1):e87138. doi: 10.1371/journal.pone.0087138 (PMC3907450; doi:10.1371/journal.pone.0087138)
Supplement: Methods S4 — Invader assay protocol. (DOCX) [file pone.0087138.s012.docx]

**Methods S4**

Invader assay protocol

1. Preparation of probe mix

The oligonucleotides for the signal probe and invader oligo were synthesized at HPLC-purification grade.

The oligonucleotides were mixed in TE buffer for each SNP locus at the following concentrations:

- 1. µM signal probe for a SNP allele detected by 6-FAM
  2. µM signal probe for a SNP allele detected by Redmond Red

0.25 µM invader oligo.

2. PCR amplification

The genomic DNA fragment carrying the SNP locus was PCR amplified. Then, the amplified DNA was denatured at 99 ˚C for 10 min.

The denatured PCR product was diluted 1/100x with dH2O for the invader assay.

3. Invader assay reaction

The solutions for the invader reaction were supplied from Hologic.

The invader reaction mixture was prepared for each sample as follows:

Probe mix: 3 µl

FRET mix: 3.5 µl

Cleavase solution: 1 µl

Diluted denatured PCR product: 7.5 µl

The prepared reaction mixture was incubated at 63 ˚C for 40 min.

The fluorescence of each solution was measured using a fluorescence microplate reader.

The conditions for measurement (excitation/emission) were 485 nm/535 nm for 6-FAM and 544 nm/616 nm for Redmond Red. For each assay, dH2O or control DNA carrying each SNP allele substituted for the DNA sample was used to measure the background or positive control.
